# Acute toxic megacolon in visceral myopathy: A rare and challenging case report with literature review

**DOI:** 10.1097/MD.0000000000043722

**Published:** 2025-08-01

**Authors:** Aladdin Al-Tartir, Lana Sbitan, Mustafa Imhaisen, Ahmad Al-Zubi, Hadi Farhat

**Affiliations:** a Department of General Surgery, Prince Hamza Hospital, Amman, Jordan; b Department of General Surgery, Urology, and Anesthesia, Faculty of Medicine, The Hashemite University, Zarqa, Jordan; c University of Balamand, Faculty of Medicine and Medical Sciences, El-Koura, Lebanon.

**Keywords:** case report, colonic diseases, intestinal pseudo-obstruction, myopathies, toxic megacolon

## Abstract

**Rationale:**

Toxic megacolon is an unusual but life-threatening condition characterized by acute dilation of the colon associated with systemic manifestations. Visceral myopathy is a rare primary motility disorder affecting smooth muscles, including the gastrointestinal system. Although it is seldom reported in the literature, toxic megacolon can present in a patient with visceral myopathy, complicating the diagnosis and management process.

**Patient concerns:**

Following CARE guidelines, we report a 25-year-old female patient who presented with 2 two-day duration of obstipation. Diagnostic work-up including, full labs, abdominal x-rays, and computed tomography scan revealed acute toxic megacolon.

**Diagnoses:**

Despite initial medical management, the patient’s condition deteriorated necessitating the need for surgical intervention. Postoperative histopathological examination revealed an underlying diagnosis of visceral myopathy.

**Interventions and outcomes:**

The patient underwent subtotal colectomy with ileorectal anastomosis and protective loop ileostomy. A multidisciplinary approach, including the involvement of gastroenterology and rheumatology teams, was crucial for optimal care and follow-up.

**Lessons:**

This case highlights the critical approach to patients with visceral myopathy presenting with acute toxic mega colon. Future research should focus on this rare but important association, to improve early recognition, diagnosis, and treatment of such complex cases.

## 1. Introduction

Toxic megacolon, a life-threatening condition characterized by extreme total or segmental non-obstructive dilation of the colon associated with systemic toxicity manifestations.^[1]^

Although this condition is marked by its rare occurrence, with an in-hospital mortality rate reaching 7.9%,^[2]^ it is important to fully understand the etiologies behind this toxic dilation for better prediction, prevention, and management.

Inflammatory bowel disease, specifically ulcerative colitis, is the most common underlying pathology associated with toxic megacolon presentation with a 10% incidence based on Greenstein et al retrospective analysis.^[3,4]^

Other related etiologies including but are not limited to: infectious causes (mostly, *Clostridium difficile*),^[5]^ ischemic colitis,^[6]^ overuse of medications that reduce bowel motility,^[1]^ and obstructive causes such as colorectal cancer, volvulus, and fecal impaction.^[7]^

Given its incompletely understood pathophysiology, acute toxic megacolon may be caused by other rare pathologies affecting colonic musculature, motility, and peristalsis, such as visceral myopathy.

Visceral myopathy is another life-threatening condition defined by bowel, bladder, and uterine smooth muscle weakness and dysfunction. Manifesting as myopathic chronic intestinal pseudo-obstruction (CIPO), obstructive uropathy, or/and uterine atony.^[8]^

Caused primarily by mutations in genes encoding different contractile proteins, gastrointestinal and bladder involvement is the most common form, which could be severe and presents at birth with intrauterine growth impairment of the colon, a condition known as megacystis–microcolon–intestinal hypoperistalsis syndrome.^[8]^

Although visceral myopathy has been linked to chronic colonic pseudo-obstruction in multiple case reports,^[9–13]^ to the best of our knowledge and our recent literature review, it has not been linked to acute toxic megacolon presentation.

In our case report, following CARE guidelines,^[14]^ we report a presentation of acute toxic megacolon in a patient with visceral myopathy, aiming to highlight this rare co-occurrence, diagnostic challenges, clinical presentation, and management approaches.

## 2. Case report

A 25-year-old female patient with no known medical comorbidities or prior surgical interventions, presented to our emergency department complaining of obstipation of 2 days duration, associated with progressive abdominal distension and intermittent colicky abdominal pain.

The pain was diffuse with increasing intensity over lower quadrants and rated 8/10 in severity affecting the patient’s daily activities. It did not radiate and was aggravated by oral intake and lying supine while fasting and sitting leaning forward partially relieved the intensity of pain. The patient also reported nausea and vomiting, especially with each oral intake, but she did not complain of a feeling of hotness, sweating, chills, hematochezia, melena, hematemesis, or change in urine or stool color.

Our patient has been suffering from chronic constipation for more than 9 months prior to this presentation, she had not received any formal diagnosis or evaluation due to financial limitations and poor healthcare access. No investigations had been performed prior to this acute presentation, and there was no known history of visceral myopathy or inflammatory bowel disease. Furthermore, the patient has never presented with prior episodes of partial or complete bowel obstruction.

As stated by the patient and her family, she did not take any regular medications and has no history of smoking, alcohol intake, or illicit drug use. The patient had no history of recent antibiotics intake for any reason.

Her family history was unremarkable, with no history of gastrointestinal disorders, visceral myopathies, inflammatory bowel disease, or toxic megacolon presentation.

On physical examination, although the patient was mildly distressed due to abdominal distension, she was hemodynamically stable at presentation, her documented vital signs were as follows: heart rate = 95 beats per minute, blood pressure = 127/67, respiratory rate = 18 breaths per minute, oxygen saturation = 95%, and a temperature of 98.4 F (36.9 °C). Her abdomen was markedly distended, with visible peristalsis noted in the upper and lower abdominal quadrants. On palpation, there was diffuse tenderness, but peritoneal signs were negative including; rebound tenderness, rigidity, and guarding. There were no palpable masses or organomegaly detected. Bowel sounds were diminished on auscultation. Digital rectal examination revealed impacted stool with solid and hard consistency, but no evidence of blood or abnormal masses. Concerning the rest of the systemic examination, cardiovascular, respiratory, and neurological examinations were unremarkable.

At presentation, laboratory investigations showed an elevated white blood cell count of 12.9 × 10^9^/L (reference range: 4–11 × 10^9^/L) with no shifting in neutrophils detected and a hemoglobin level of 14.2 g/dL (reference range: 12–16 g/dL). Serum electrolytes revealed mild hypokalemia and hyponatremia, while renal and liver function tests were within normal limits. C-reactive protein (CRP) and erythrocyte sedimentation rate levels were significantly elevated. On the other hand, arterial blood gas analysis demonstrated normal acid-base balance (refer to Table 1).

**Table 1:** Clinical and laboratory parameters.

Parameter	Day 1	Day 2	Day 3	Day 4	Day 5	Day 6	Day 7	Day 8	Day 9	Day 10	Reference range
Temperature	36.9	37.5	36.5	37.0	37.2	36.8	36.8	37.0	37.1	37.0	36.0–37.5 °C
Heart rate	95	100	135	85	90	98	80	75	72	88	60–100 beats/min
Blood pressure	127/67	120/75	106/74	110/60	116/65	122/75	125/70	123/65	120/75	118/70	100–140/60–90 mm Hg
Respiratory rate	18	20	28	18	17	15	20	15	14	15	12–20 breaths/min
WBC count	12.9	14	16	22	22	19	15	12	10	9.4	4–11 × 10^9^/L
Hemoglobin	14.2	14.0	12.3	11.0	10.5	11.2	11.0	11.0	10.5	10.4	12–16 g/dL (female)
Platelet count	350	345	420	400	330	300	375	250	346	370	150,000–400,000/µL
Serum sodium	140	142	148	137	136	135	135	133	136	140	135–145 mmol/L
Serum potassium	4.0	3.5	3.0	3.2	3.1	3.5	3.5	4.0	4.2	4.5	3.5–5.0 mmol/L
Serum creatinine	0.9	0.7	1.3	0.6	0.7	0.8	0.9	0.9	0.8	0.9	0.6–1.1 mg/dL (female)
CRP	150	140	143.96	240	221	110	97	63	15	23	<5 mg/L
Arterial blood gas	7.4, 24	7.36,22	7.24,27	7.3, 30	7.38, 24	7.4, 22	7.42, 24	7.4, 24	7.38, 22	7.4, 25	pH: 7.35–7.45, HCO₃⁻: 22–26

CRP = C-reactive protein.

**Figure 1.**
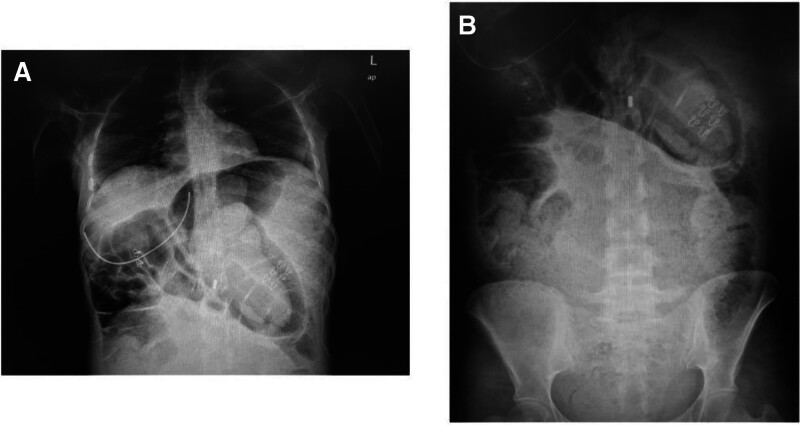
Abdominal X-ray at presentation demonstrating features of toxic megacolon. (A) Abdominal X-ray revealing marked colonic dilation with a loss of haustrations, highly suggestive of toxic megacolon. No evidence of free air to indicate perforation. (B) Significant colonic dilation is evident, with associated fecal impaction. A nasogastric tube is visible in the upper abdomen. These findings further support the diagnosis of toxic megacolon with intestinal obstruction.

Imaging studies included an abdominal x-ray in erect and supine positions, which showed marked colonic dilation with loss of haustrations, suggestive of toxic megacolon and no signs of free air indicating perforation (refer to Fig. 1). Computed tomography scan with intravenous (IV) and oral contrast confirmed colonic dilation (rectum and sigmoid colon diameter measured approximately 10 cm, while cecum measured 4.8 cm), focal fecal material in the rectum (fecaloma), and no signs of pneumoperitoneum.

The diagnosis of toxic megacolon was suspected based on the patient’s clinical presentation imaging and laboratory investigations, with reference to Jalan toxic megacolon diagnostic criteria.^[15]^

With the absence of surgical indications, initial treatment involved supportive therapy. Our patient was admitted to the surgical intensive care unit. She was kept NPO (nil per os), a nasogastric tube was inserted for decompression, full septic work-up including blood cultures, urine analysis and culture, and chest imaging was conducted, IV broad-spectrum antibiotics (Metronidazole and Ceftriaxone) were given and IV fluid was administered for adequate hydration and electrolytes correction. Labs (including complete blood count, electrolytes, and kidney function tests) and abdominal films were repeated every 12 hours to monitor the patient for possible deterioration.

Infectious causes of toxic megacolon were considered. However, stool studies (especially, *C difficile* toxin assay) returned negative. In addition, septic work-up done at presentation returned negative with no source of infection identified.

**Figure 2.**
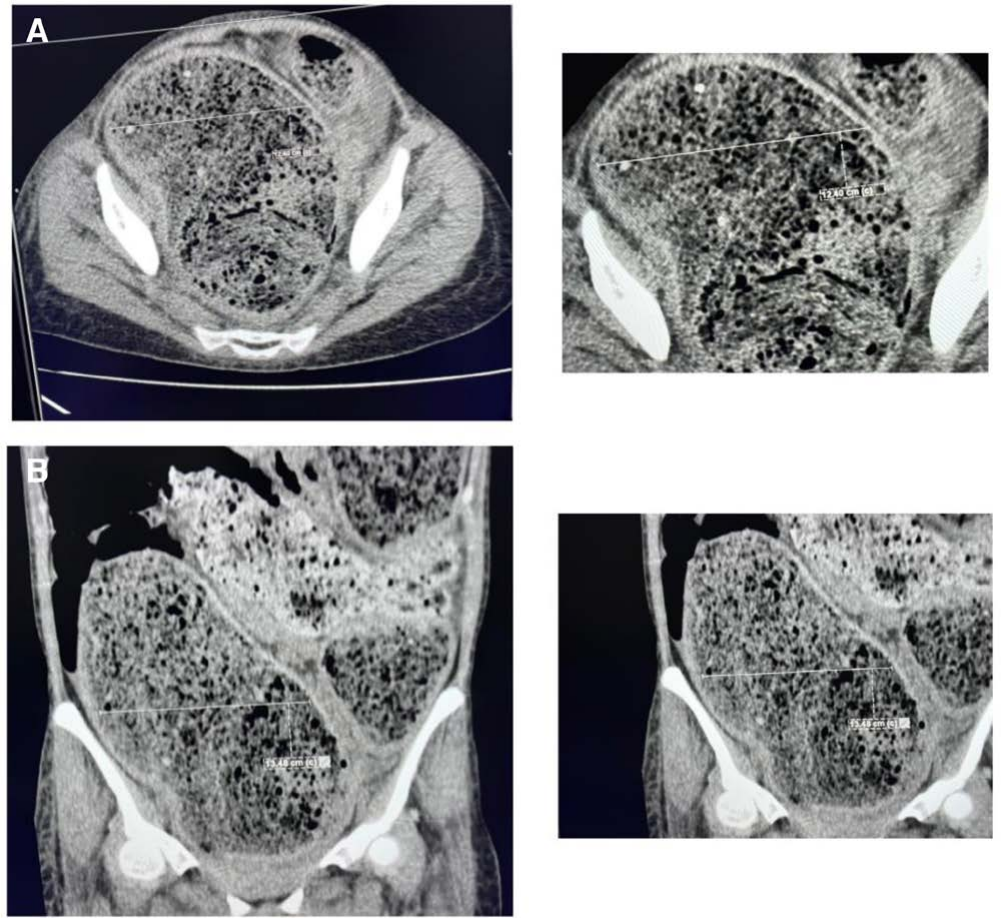
CT imaging demonstrating extensive colonic dilatation in toxic megacolon. (A) Axial CT scan showing marked dilatation of the sigmoid, descending, and transverse colon, with the largest diameter observed in the sigmoid colon (approximately 12 cm), along with fecal impaction. (B) Coronal CT scan further illustrates the extensive colonic dilatation and fecal accumulation, indicative of toxic megacolon. CT = computed tomography.

On day 3 of conservative management, the patient’s condition worsened despite supportive measurements taken. Her vital signs were as follows: heart rate = 135 beats per minute, blood pressure = 106/74, respiratory rate = 28 breaths per minute, oxygen saturation = 94%, and a temperature of 97.7 F (36.5 °C). Tense abdominal distension persisted and was associated with increasing discomfort. Repeat imaging studies revealed further extensive dilatation of sigmoid, descending, and transverse colon (the largest transverse diameter was seen at the sigmoid colon measuring about 12 cm) with impacted fecal material (Fig. 2). Laboratory inflammatory markers including CRP and erythrocyte sedimentation rate, remained elevated. The patient developed marked leukocytosis, neutrophils shifting (16 × 10^9^/L, reference range: 4–11 × 10^9^/L), drop in hemoglobin (12.3 g/dL (reference range: 12–16 g/dL)), and CRP level of 143.96 mg/L (reference range 0–5 mg/L) (refer to Table 1). Taking into consideration the risk of further deterioration, potential of perforation, and sepsis, the surgical team decided to proceed with surgical intervention.

**Figure 3.**
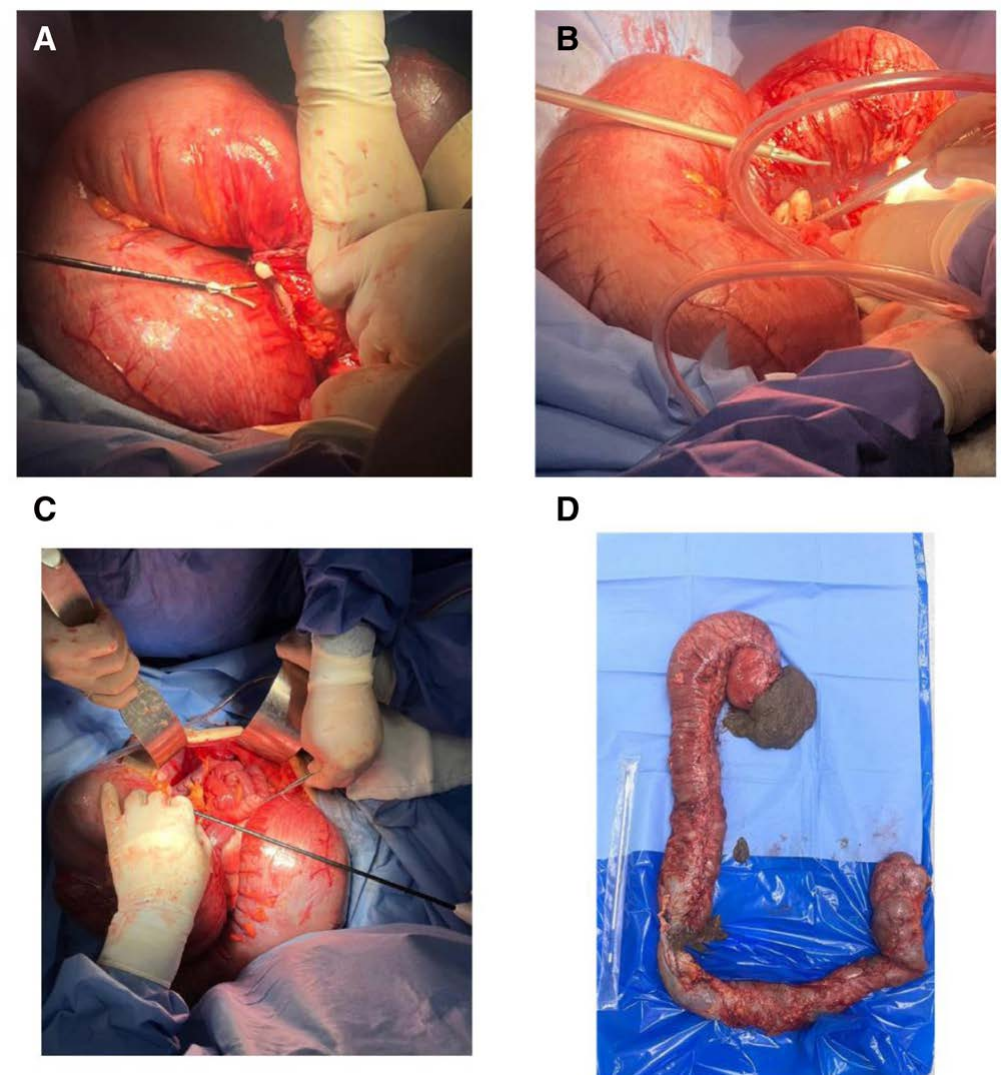
Intraoperative findings of dilated colonic segment in toxic megacolon. (A–C) A massively dilated colonic segment intraoperatively, with a thin and distended bowel wall, consistent with toxic megacolon. (D) Presents the resected colonic segment post-excision, highlighting its abnormal dilation and loss of normal haustration.

Emergent subtotal colectomy with ileorectal anastomosis and protective loop ileostomy were performed. Intraoperative findings showed gross dilatation of the entire colon, with the maximum transverse diameter measuring approximately 20 cm. Colonic serosa appeared inflamed, but there was no gross evidence of perforation, necrosis, or adhesions. Additionally, no intra-abdominal collection or abscess was noted (Fig. 3).

Postoperatively, the patient was closely monitored in surgical intensive care unit for signs of infection or sepsis. Nutritional support was provided through the parenteral route with gradual conversion to enteral feeding as tolerated.

The patient was kept on antibiotics treatment from day 1 of admission and continued until hospital discharge, in alignment with institutional sepsis management protocols. The total duration of antibiotic therapy was 10 days.

**Figure 4.**
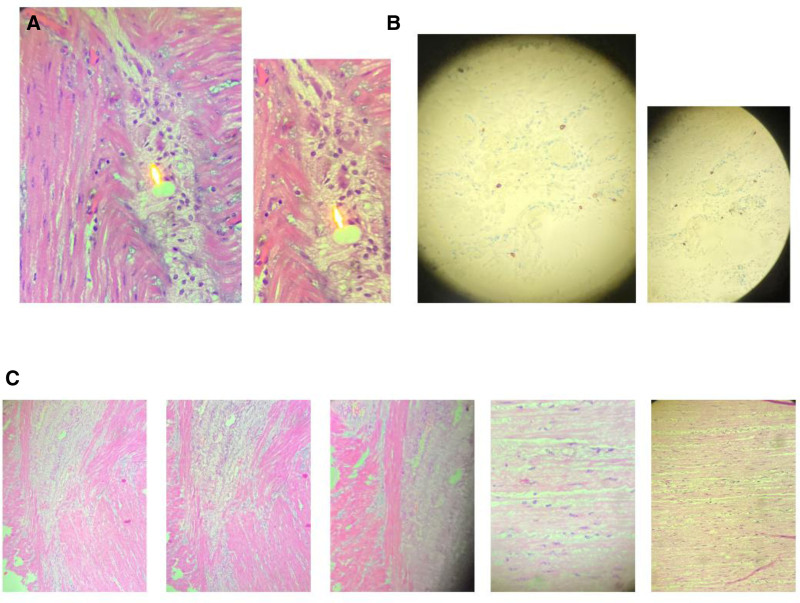
Histopathological examination of resected colonic segments. (A) Mild neural hypertrophy with preserved ganglion cells in both Auerbach and Meissner plexuses. (B) Immunostaining for CD117 (Cajal cells) is adequate, showing a normal distribution. (C) H&E-stained section showing degenerated smooth muscle fibers within the muscularis propria, with interstitial fibrosis and loss of normal architecture. Vacuolated smooth muscle cells are evident, along with scattered nuclear changes.

A resected colon specimen was sent for histopathological examination through which confirmation of motility disorder (most likely visceral myopathy) was made (Table 2 shows full details related to histopathology results). Furthermore, Figure 4 represents histopathological examination images revealing marked thinning and fibrotic replacement of the muscularis propria, with vacuolated and degenerated smooth muscle fibers, consistent with a diagnosis of visceral myopathy. No ganglion cell loss or inflammatory infiltrate was observed in the myenteric plexus. Given the patient’s underlying condition, a referral to the gastroenterology and rheumatology teams was made. To rule out autoimmune and connective tissue disorders, she underwent full investigations (Table 3).

**Table 2:** Histopathological findings of the resected colon.

Histopathological findings	
Gross description	- Colectomy specimen measuring 120 cm in length. - The site of perforation is located 60 cm from the distal margin. - The specimen is not prepared and is filled with fecal material. - An attached appendix is observed, measuring 5 cm in length. - Upon opening, no tumor is identified. - Grossly dilated, flat mucosa.
Microscopic description	The provided sections show: - Full-thickness colonic tissue with predominantly preserved architecture. - Foci of goblet cell hyperplasia and crypt hypertrophy. - Areas of mucosa with goblet cell depletion. - The lamina propria exhibits diffuse pigmented histiocytes (melanosis coli) and mild, mixed inflammatory infiltration. - The muscularis propria shows focal fibrosis, primarily in the circular layer, with thinning, muscle degeneration, and vacuolation. - The subserosa reveals focal fat necrosis. - Mild neural hypertrophy with unremarkable ganglion cells in both Auerbach and Meissner’s plexuses. - Two small incidental reactive lymph nodes are noted. - No evidence of malignancy.
Immunostaining	- Sections from the appendix are unremarkable. - Immunostaining for CD117 (Cajal cells) is adequate, showing a normal distribution.
Surgical pathology diagnosis	- Motility disorder, most likely visceral myopathy. - Melanosis Coli.

**Table 3:** Rheumatological tests and their results.

Test name	Result	Range
Rheumatoid factor	<20	≤30
CCP IgG	0.6	≤4.99
ENA (extractable nuclear antigens)		
Double-stranded DNA (dsDNA)	Negative	Ref: Negative
Nucleosomes	Negative	Ref: Negative
Histones	Negative	Ref: Negative
Smith antigen (SmD)	Negative	Ref: Negative
Proliferating cell nuclear antigen (PCNA)	Negative	Ref: Negative
Ribosomal protein (P0)	Negative	Ref: Negative
Ro 60 kDa (Ro60)	Negative	Ref: Negative
Ro52 kDa (Ro52)	Negative	Ref: Negative
La (SS-B)	Negative	Ref: Negative
Centromere protein B (CENP B)	Negative	Ref: Negative
SC170	Negative	Ref: Negative
U1 small nuclear ribonucleoprotein (U1-SNRNP)	Negative	Ref: Negative
M2	Negative	Ref: Negative
Jo-1	Negative	Ref: Negative
Polymyositis-scleroderma (PMSc1)	Negative	Ref: Negative
MI-2	Negative	Ref: Negative
Ku	Negative	Ref: Negative

Over the course of 3 months and through multiple follow-up visits to the clinic, our patient reported gradual improvement of her symptoms and no signs of postoperative complications. She was also following up with the gastroenterology team regularly to monitor GI function and motility. This form of multidisciplinary approach guarantees comprehensive care.

Six-weeks postoperatively, colonoscopy was performed which showed a normal mucosal appearance, ruling out structural or inflammatory causes of dysmotility (e.g., IBD), further supporting a primary motility disorder.

## 3. Discussion

Toxic megacolon is a well-documented phenomenon related to inflammatory or infectious causes. Nevertheless, its occurrence due to colonic myopathies and primary motility disorders is rare and seldom reported in literature. Our case report demonstrates the diagnostic challenges behind such a presentation and emphasizes the importance of considering such conditions, including visceral myopathy in patients presenting with toxic megacolon.

The pathophysiology behind toxic megacolon is not entirely understood. However, one of the proposed mechanisms highlights the role of mucosal inflammation in the release of inflammatory mediators and inducible nitric oxide, which contribute to colonic muscle dilation and neurogenic dysfunction.^[16]^ Furthermore, this toxic dilation leads to weakening of the colonic wall, breaking of the mucosal barrier, and promoting bacterial translocation.^[1,17]^

Although inflammatory bowel disease was considered in the differential diagnosis, as it is considered a significant related condition to toxic megacolon, the absence of typical clinical features and the inability to perform preoperative colonoscopy due to acute deterioration limited early confirmation. A postoperative colonoscopy at 6 weeks showed normal mucosa, effectively ruling out IBD as an underlying cause in our case.

Visceral myopathy has been characterized by dysfunction in smooth muscles. As smooth muscles depend in their function on contractile apparatus cytoskeletal proteins for their optimum function, mutations in genes encoding those proteins (including but not limited to; ACTG2, ACTA2, MYH11, MYLK, LMOD1, MYL, and FLNA), lead to primary motility disorder especially affecting gastrointestinal muscles.^[8]^

Although genetic testing is essential in establishing a definitive diagnosis of primary visceral myopathy, genetic sequencing was not available in our setting, and the patient’s family declined external testing. As such, the diagnosis in this case relied on classical histological features and the exclusion of alternative etiologies.

While familial visceral myopathy has been reported in association with genetic mutations, the absence of any family history of gastrointestinal disorders in this patient supports the likelihood of a sporadic form.

CIPO, another rare entity that has been linked to visceral myopathy and was categorized as the main GI manifestation of this condition.^[8]^

Presenting mainly as intermittent episodes of nausea, vomiting, abdominal pain and distension.^[18]^ Reviewing the literature, we identified 3 case reports linking visceral myopathy with CIPO,^[9,10,12]^ (refer to Table 4 for full description of each case report). Only 1 case out of the 3 reported acute presentation of megacolon.^[9]^

**Table 4:** Summary of reported cases of visceral myopathy associated with chronic intestinal pseudo-obstruction.

Case	Country	Age/sex	Presentation	Diagnostic findings	Management	Outcome
Hong et al^[9]^	Korea	31-yr-old/female	Refractory constipation accompanied by megacolon	An abdominal CT revealed: - Large luminal dilatation of the sigmoid colon with impacted stool. - No obstructing lesion or luminal dilatation of the rectum	Surgical: Total colectomy with an ileorectal anastomosis	Patient was discharged without any intra-abdominal symptoms.
Pal et al^[12]^	Saudi Arabia	6-yr-old/male	Episodes of intermittent abdominal distension, bilious vomiting, and constipation of 2 years’ duration.	Plain abdominal radiographs and contrast barium swallow with follow-through showed: - Dilated loops of intestine throughout the abdomen beginning at the duodenum.	Surgical: Laparotomy was performed (antegrade and retrograde decompression of the stomach and rectum, respectively).	Patient was diagnosed with Sanjad-Sakati syndrome or hypoPTH-retardation-dysmorphism syndrome.
Philip et al^[10]^	India	62-yr-old/female	Acute intestinal obstruction	X-ray abdomen showed: - Grossly dilated small bowel loops with multiple air fluid levels. A stat computed tomography (CECT) Scan of the abdomen and pelvis showed: - Grossly dilated jejunal and ileal loops (10 cm) with multiple air fluid levels and transition point at the terminal ileum with abrupt tapering, suggestive of small bowel obstruction.	Surgical: Emergent operative exploration (an enterotomy was made in the distal ileum to decompress the grossly dilated small bowel and diversion loop ileostomy).	Patient developed prolapse of the ileostomy loop 10 mo later. She underwent surgical repair and was doing well on follow-up.

CT = computed tomography.

However, with reference to the pathophysiology of both conditions understood so far, both visceral myopathy and toxic megacolon share a common basic mechanism of severe dysmotility and impaired gut wall neuromuscular function. The fundamental disruption of coordinated peristalsis in both conditions highlights a possible overlap where long-standing primary motility disorders increase the predisposition of patient to secondary colonic dilation and failure of peristalsis as seen in toxic megacolon.

It is important to recognize that visceral myopathy primarily affects the small intestine and colon, and its diagnosis does not require evidence of esophageal involvement. While esophageal motility disorders are typically assessed using high-resolution manometry, this investigation was not indicated in our patient due to the absence of upper gastrointestinal symptoms and the colonic-dominant presentation.

Among the 3 reported cases, only Hong et al presented a patient with megacolon without systemic toxicity manifestations who had long-standing refractory constipation.^[9]^ Compared to our patient, the evidence of systemic toxicity made the diagnosis of toxic megacolon more plausible. Furthermore, our patient had a more acute presentation over a 9 months period compared to Hong et al patient who had been suffering from constipation and abdominal pain over a 5 years duration prior to presentation with worsening of symptoms.^[9]^

Hong et al, Pal et al, and Philip et al reported diagnosis of CIPO in their patients with prolonged history of constipation.^[9,10,12]^ Conservative suppurative management was not effective in all reported cases, similar to our case, as all 3 patient underwent different kinds of surgical management (total colectomy with an ileorectal anastomosis, laparotomy with Antegrade and retrograde decompression of the stomach and rectum and distal ileal enterotomy with diversion loop ileostomy, respectively).^[9,10,12]^

In the only reported pediatric case, an underlying diagnosis of Sanjad–Sakati syndrome was confirmed, which underscores the importance of a full multidisciplinary approach to rule out syndromic or connective and autoimmune disorders.^[12]^ Our patient as demonstrated in Table 3 underwent full investigations and clinical assessments for possible correlations, especially with scleroderma or autoimmune diseases.

In general, similar to our reported case, the outcome and prognosis of patients in the documented cases is good with patients discharged without intra-abdominal symptoms and on prolonged suppurative nutritional measurements, including multivitamins supplements for possible malabsorption.^[9,10,12]^

Although diagnosis of toxic megacolon in patients with myopathic bowel presents a challenge for clinicians due to symptoms overlap. Primary motility disorders should be added to the underlying differential diagnosis of toxic megacolon in addition to inflammatory and infectious causes. A comprehensive approach through clinical assessment and thorough history taking, imaging studies, and laboratory investigations will help to identify such cases and avoid delay in treatment, improving prognosis and outcome.

Management approach for patients with visceral myopathy and toxic megacolon should follow the general guidelines of toxic megacolon management,^[9,10,12,19]^ with early surgical intervention in case conservative treatment failure or high risk of perforation.

It is important to emphasize the importance of multidisciplinary approach, including surgical, medical, and nursing expertise for optimum patient care. Even on follow-up of patients postoperative management, the gastroenterology team should be involved with the surgical team in monitoring patient condition.

Clinically, caution should be taken in patients with myopathic bowel presenting with acute abdomen, as timely diagnosis, monitoring, and management are necessary to prevent complications.

Further research is needed to explore this rare association for better understanding of underlying pathophysiology, diagnostic evaluation, and management.

## 4. Conclusion

This case report highlights the diagnostic challenges of acute abdomen in patients with underlying motility disorders, emphasizing the need for early diagnosis, multidisciplinary approach, and timely intervention whether medical or surgical. Management of such complex cases requires further research to support early recognition and prevention of life-threatening complications.

## Acknowledgments

We extend our gratitude for all healthcare professionals who were involved in the management of our patient. Special thanks for radiology, pathology, gastroenterology and rheumatology teams.

## Author contributions

**Conceptualization:** Lana Sbitan.

**Methodology:** Lana Sbitan.

**Visualization:** Lana Sbitan.

**Writing – original draft:** Lana Sbitan, Mustafa Imhaisen, Ahmad Al-Zubi.

**Writing – review & editing:** Aladdin Al-Tartir, Lana Sbitan, Hadi Farhat.
